# Evaluation of Accuracy and Performance of isCGM Sensors during Experimentally Induced Glucose Excursions. Comment on Moser et al. Performance of the Intermittently Scanned Continuous Glucose Monitoring (isCGM) System during a High Oral Glucose Challenge in Adults with Type 1 Diabetes—A Prospective Secondary Outcome Analysis. *Biosensors* 2021, *11*, 22

**DOI:** 10.3390/bios12030152

**Published:** 2022-03-01

**Authors:** Wiebke Jessen, Alexander Seibold

**Affiliations:** Abbott Diabetes Care, 65205 Wiesbaden, Germany; wiebke.jessen@abbott.com

A 2021 publication in *Biosensors* from Moser and colleagues [1] reported on the accuracy of the FreeStyle Libre^®^ intermittently scanned continuous glucose monitoring (isCGM) system at different rates of change in plasma glucose (RCPG), during two oral glucose tolerance tests (OGTT) in 19 adults with type 1 diabetes (T1D), following 12 and 36 h fasting periods. They concluded that the FreeStyle Libre system is accurate when compared to reference plasma glucose during OGTT glycemic challenge, but that its performance was dependent on the RCPG and that it overestimated glucose during periods when reference blood glucose values were in hypoglycemia.

The performance of CGM systems at different glucose concentrations and rates of change is essential to know [2]. However, this objective information must be reported in a timely fashion, since all CGM systems are constantly evolving to provide an ever more accurate correlation between the measurement of glucose in interstitial fluid (ISF) and contemporaneous blood glucose values. We must point out that the report by Moser et al. reflects the measurement of ISF glucose using the FreeStyle Libre sensor available at the point of first enrolment of subjects in their study in June 2018. However, by January 2021 when the study was published, the FreeStyle Libre system had already been updated with a new algorithm with a superior accuracy and reduced time lag [3]. Moser et al. report that the FreeStyle Libre system in their study had a mean absolute relative difference (MARD) of 18.8% at low glucose <70 mg/dL compared to reference blood samples, based on only 6 paired samples from 19 subjects. Although the most recent algorithm for the FreeStyle Libre sensor does not report the MARD for readings exclusively <70 mg/dL, it has a mean absolute difference (MAD) of 6.8 mg/dL for readings 51–80 mg/dL and a MAD of 8.8 mg/dL for readings 40–50 mg/dL, based on 1213 and 62 paired readings, respectively, with 98.4% of readings <70 mg/dL within ±20 mg/dL of the reference blood glucose sample [3]. In submitting their data for publication, they unwittingly reported on the legacy performance of a glucose monitoring system, rather than the actual performance of the system that is available for daily diabetes management.

Moser and colleagues also reported that the FreeStyle Libre sensor overestimated glucose during periods when reference blood glucose values were in hypoglycemia. In fact, concurrence data for the FreeStyle Libre sensor indicates that the algorithm tends to underestimate glucose levels in the low-glucose zone, which has the effect of alerting users to potential hypoglycemia when actual blood glucose may still be in the euglycemic range. Similarly, a potential source of difference between ISF sensor readings and reference blood glucose at higher RCPG is the time lag between glucose change in blood and subsequently in ISF. For the earlier-generation algorithm in the study by Moser et al., the time lag was 4.5 min (±4.8) min [4]. The most recent algorithm has seen this reduced to 2.4 min (±4.6), further increasing the responsiveness of FreeStyle Libre sensors at high RCPG [3].

We accept the need for an independent review of the accuracy and performance of widely used glucose monitoring technology. However, it is important that the outcomes of such assessments maintain their currency in this dynamic area of diabetes monitoring and management. This should also be a criterion for independent peer review of glucose sensor accuracy data prior to publication, since the most recent accuracy data is readily available from CGM product manufacturers in peer-reviewed literature and product handbooks. It would make sense in this context to require all such manuscripts to detail in the methods section the version of the glucose data management software that was applicable during the study. Where this does not match the most recent version, a clear decision can then be made on the relevance and value of the outcomes.
